# An Inquiry into the Nature and Properties of the Blood in Health and Disease

**Published:** 1835-04-01

**Authors:** 


### An Inquiry into the Nature and Properties of the Blood, in Health and Disease. By the late *Charles Turner Thackrah*. A new and enlarged Edition, arranged and revised by *Thomas G. Wright*, M.D., to which is prefixed a Biographical Memoir of Mr. Thackrah. London, 1834.

The industrious author of this work, already rather favourably known to the medical public by the first edition of it, published in 1819, as well as by some other medical essays, did not, unfortunately, live long enough to complete and superintend the present posthumous edition. The author's papers, from which the work as we now have it has been compiled, were committed by his widow to the care, revision, and arrangement of Dr. Wright, and certainly, if we may judge from a mere inspection of the matter, the task was not one of the most agreeable. The volume is dedicated to Sir A. P. Cooper, under whose auspices the first edition was published, and commences with a biographical memoir of the author, from which we learn that he had been a long time labouring under very delicate health, whilst preparing this second edition, which death prevented him from completely finishing. This of course accounts satisfactorily for the unconnected and detached form in which many of the observations in the book present themselves. The day is now gone by, when the importance of such a subject as "an Inquiry into the Nature and Properties of the Blood, in Health and Disease," could be for a moment called in question. To detail the general properties of the circulating mass, its several physical and chemical habitudes, its peculiarities, as depending on the different parts of the system in which it is found, as well as in the different classes of animals—to point out the various modifications and changes produced in it by disease, as also the therapeutical indications thence afforded to the physician, must undoubtedly strike the most cursory observer as matters of no ordinary moment. When we consider what were the medical doctrines which prevailed for so long a time in the schools, whereby all morbid phenomena were referred either to the influence of the nervous system, or to the agency of the solids, we cannot feel much surprize that the constitution of the blood, and of the other fluids of the body, and the changes produced in them by disease, were almost entirely disregarded. That the humoral pathology led to very many erroneous ideas regarding the nature and treatment of disease, no one will attempt to deny. There was, we know, a time when every disease was referred to acidity, or alkalescence of the blood, or of some of the other fluids of the body. We are also well aware that, when such theories were taken up, the only legitimate mode of investigating the nature and seat of disease was altogether abandoned, and gratuitous hypothesis usurped the place of attentive observation of facts, and rigorous induction from them. This error being in time detected, the entire humoral pathology fell into almost universal neglect, and medical men, running from one extreme into another, embraced the diametrically opposite doctrine of exclusive solidism. But now that the Baconian method of philosophizing has been applied to medicine, as well as to the other branches of physical science, when not only every organ and tissue, but every fluid of the body, is subjected to experiment, it was not difficult to foresee that the theory of exclusive solidism could not hold its ground much longer, numerous cases of disease continually presenting themselves, of which it was found totally incapable of affording any satisfactory explanation. When we consider the similarity subsisting between the proximate principles of the blood and the solids of the body, and the close physiological connexion which also subsists between them, it will be no easy matter to conceive how disease could exist to any amount in the solids, without the blood being also affected more or less, nor how the nature and constitution of the blood could be materially altered, without such alteration producing a reflected alteration in the state of the solids. And certainly, if complexity of formation be a sufficient ground whence to infer liability to morbid change, such liability will be readily admitted to exist in the blood; and hence the necessity and importance of a work purporting, as the present does, to detail those various changes and modifications, must be manifest. How far our author has succeeded in helping to supply such a desideratum in medical literature, we shall now proceed to consider.

After enumerating in the preface the various difficulties and obstacles which stand in the way of this inquiry, and their causes, we meet the following modest and unpretending observations.

"To clear away these obstacles has been one object of my attention, and if in this only I have been successful, science will be benefited. The labourer who removes the rubbish on the site of a projected building, raises not indeed the structure, but in preparing the way for the more able workman, he takes an office, though less respectable, yet not, perhaps, less useful." 22.

Again, to the same effect, we read as follows:

"Whatever opinion may be entertained of the success of my researches, of the mode in which they have been conducted, or of the conclusions to which they have led, I lay claim to fairness of intention and honesty of detail. Unbiassed by prejudice, unshackled by preconceived notions, I have impartially stated the individual results of my experiments, and noticed every regular or casual discordancy. It has been my aim rather to ascertain facts than to support opinions—to study the œconomy of nature, rather than to fetter her with conjectural or inconsistent theories." 23.

Having briefly described the course of the circulation, and the changes which the blood undergoes therein, the several secretions, &c. derived from it, he says—

"The formation of the blood is a process imperfectly understood. We know that food is digested and chyle formed; that this milky fluid is carried into the blood; that other liquids, thinner and in larger quantities, are also absorbed, and rapidly transmitted to the circulatory system; and we believe that these are the two principal sources of supply. Whenever sanguification be completed, the materials seem long in the course of preparation. Chyle taken from the lacteals coagulates, and the fluid in the absorbents approaches in character to the serum of the blood." 29.

After giving the several and widely-differing estimates of the quantity of blood in the body, as furnished by different physiologists, some of whom (Keill) stating it to be 100 lbs.—others estimating it at not more than 8 lbs.; Haller at from 28 to 30, and Young at 40, he says—

"The wide difference in these estimates seems to depend on the want of such data as are requisite for accurate research. It is true, indeed, that the contents of the several arteries and veins may be subjected to calculation, but of the blood circulating in the capillaries no accurate estimate can be formed. When we reflect on the minuteness of these capillaries, on the universality of their distribution, and on the large proportion which they constitute of muscle and other solids—when we remark, also, the red colour which the flesh of a slaughtered animal retains, we must refuse to admit any estimate formed from the quantity of blood drawn off in fatal hemorrhage. The blood, moreover, cannot be obtained in toto without the admixture of its secretions. If we collect the blood of a slaughtered animal, our estimate is invalidated by the changes which take place during the period, and especially by the water, or a serous fluid, which is largely absorbed into the circulation during hemorrhage." 30.

We must object, in toto, to an opinion contained in the above extract, viz. that the red colour of muscle depends on blood, either contained in its vessels or extravasated through them. The illustrious Bichat set that question at rest long since.

The various estimates regarding the *size* of the globules of the blood are next presented to us. Some physiologists stated them to be the $\frac{7}{1500}$ of an inch in diameter, others $\frac{1}{4000}$ths; Wollaston computed them at $\frac{1}{4900}$ths; Young $\frac{1}{6060}$ths; whilst Sir E. Home and M. Bauer estimated the diameter of a globule, devoid of its colouring matter, at $\frac{1}{2000}$ths. Next the *form* of these particles has been the subject of dispute. Leuwenhock represents the globules as circular when at rest, and elliptical when in motion; and states that each principal globule consists of six minor and separable globules, each of which again consists of six other smaller globules. Hunter considered these particles to be globular and equal in size in the same animal. Blumenbach states them to be globular when at rest, and oval when in motion. Raspail represents them as varying in size in different vessels of the same individual. After describing the separation of the blood when taken from the body into the *serum* and *crassamentum*, he takes up the remainder of the first chapter in combating Mr. Hunter's theory of the vitality of the blood. From the manner in which our author argues the matter in this and in a subsequent chapter, we must say that he was entirely out of his element, when he attempted to venture on metaphysical disquisitions.

With respect to the *specific gravity* of healthy blood, our author averages it at 1041;—he estimates the *solid contents* at nearly one-fifth of the entire mass. M. Le Cann, who has given an analysis of the blood, in the Journal de Pharmacie for Sept. 1831, gives the proportion of water as 780·145 in 1000. The average specific gravity of healthy *serum* our author states to be from 1020 to 1030.

"*Serum* is principally composed of albumen and water; but it contains also carbonate, sulphate, and muriate of soda; muriate and sulphate of potass; phosphates of soda and of lime, and a little impure acetate of soda. Serum largely absorbs carbonic acid. On agitating a small quantity in three inches of carbonic acid gas, one inch quickly disappeared. The proportion of albumen in serum is, on the average of our experiments, 42 in 1000: that of saline matters less than 1 in 1000. Serum is said to contain a free alkali, which, according to Berzelius, Marcet, and Bostock, is soda." 40.

"The solid contents left by evaporating serum are stated by Dr. Bostock to be 12 per cent. My examination presents a different result: 4·4 per cent. is the average of our experiments on the serum of healthy blood. My observations, moreover, shew that the last effusion of serum from the crassamentum contains a much greater proportion of albumen than the first.

A temperature of 150°—160° quickly reduces serum to a coagulum, which is principally albumen. From coagulated serum a small quantity of fluid may be pressed, which has unfortunately obtained the name of *serosity*. It is water holding in solution about one-fiftieth part of albuminous matter, with a considerable proportion of alkaline salts." 41.

After stating the *crassamentum* or clot to consist of red particles, fibrine, and a very important ingredient which has been overlooked or but slightly noticed, but which constitutes the largest proportion, namely, albumen, he next comes to consider the colouring matter of the blood. Various artificial means have been proposed by different chemists for the separation of this from the other ingredients. To this constituent of the blood continental writers have given the name *hæmatosine*. Berzelius and Engelhart have demonstrated iron to form the basis of this substance. The state in which iron exists in the blood, whether as a mere oxide, or in combination with an acid, is a matter as yet not satisfactorily ascertained. Hæmatosine is the only constituent of the blood not found in any of the solids or secretions of the body.

We next come to the consideration of another constituent of crassamentum, namely, the *fibrine*. This may, in general, be obtained by enclosing crassamentum in a linen bag, and by pressing and washing the mass, till a stringy substance alone appears. This is fibrine, combined with a small proportion of oil, albumen, and saline matter. The elements of fibrine are azote, carbon, and oxygen;—azote is contained in fibrine in a larger proportion than in any other animal substance.

" The proportion of fibrine in blood is stated by *Berzelius* at ·75 to 1000, but *Whiting*, from his experiments, concludes it to be from 1 to 2 in 1000. Our experiments give an average of 2·8 in 1000. The sp. gravity of fibrine, according to Davy, varies from 1046 to 1060. Prout and others have considered it to be less than that of serum, ' since fibrine usually swims in the serum.' The simple experiment, however, of dropping some *pressed* fibrine into serum, shews at once the sinking of the solid, and the consequent error of this opinion."—" In the circulatory blood fibrine is either held in solution, or exists, according to the observation of *Bauer*, in the state of very minute white globules. It is well known to form the basis of muscle; and so nearly does it approach to organized matter, that the galvanic aura increases its contraction. It affords the framework of the body, and that also of the preternatural structures resulting from disease. Coagulable or plastic lymph is a combination of fibrine and albumen." 47.

The upper surface of the crassamentum is generally observed to be of a much more florid colour than the lower: this has been said to be caused by the atmosphere, which removes the carbon from all that part of the clot under its influence. Dr. J. Davy rejects this opinion, and will have it that the air produces this effect by altering the specific gravity of the hæmatosine, which then subsides in the clot. In Appendix II. of the work, an experiment (12) is adduced intended to refute Dr. Davy's opinion, but which we are disposed to consider as corroborative of it; certainly it cannot be looked on as at all tending to weaken it. According to Dr. Stephens the florid colour of the clot is to be referred to saline ingredients contained in the serum.

The remaining constituents of the blood are " an oily substance named by M. Denis " GRAISSE PHOSPHUREE ROUGE," and " GRAISSE PHOSPHUREE BLANCHE," noticed by *Berzelius* and other chemists.

" It appears to be identical with the Matière cerebrale rouge, and the Matière cerebrale blanche, which Vauquelin discovered in the brain. In addition to the ordinary elements of animal matter, the oil is found to contain phosphorus and sulphur. It is obtained by exposing the dry contents of blood to the action of æther at a moderately high temperature, and under strong pressure; or by boiling them in alcohol. On gradually cooling the solution the *graisse* subsides." 49.

Another of the ingredients of blood is the MUCO-EXTRACTIVE MATTER of *Marcet;* the IMPURE LACTATE OF SODA of *Berzelius;* the OZMAZOME of *Denis*. This may be procured from the blood by digestion in cold alcohol. It possesses the properties of the substance, which Thenard extracted from muscular fibre, and to which he gave the name of Ozmazome.

Another ingredient discovered in the blood by *M. Denis* is CRUORINE, which is solid, colourless, and transparent. This is soluble in water, particularly cold water, and insoluble in alcohol and æther. It is most readily procured from fibrine, " by boiling this substance dried and pulverized for five to ten minutes, in forty or fifty times its weight of water. The mixture is then to be filtered and slowly evaporated; and when the residue has been washed with hot alcohol, to remove the oil, pure cruorine will remain at the bottom of the vessel." *Ibid.*

The editor gives from himself an analysis of the *halitus* of the blood, which, according to Fourcroy, consists almost entirely of aqueous vapour, holding in solution a minute portion of animal matter; which *Bondet* supposes to be a volatile acid substance, analogous to the " graisse phosphuree" of Chevreuil and Denis. Haller considered this identical with the matter of perspiration. It is found to be of a more pungent, and of a ranker odour in males than in females, and is said to be wanting in eunuchs and old people, and for this reason is supposed to be connected with virility.

The results of the author's experiments on healthy blood, with respect to its three chief constituents, are as follows:—in 1000 parts—

| | |
|---|---|
| Water | 796·55 |
| Hæmatosine and albumen | 200·1 |
| Fibrine | 2·8 |
| Dry contents of serum (saline) | ·55 |
| | 1000·00 |

After offering some conjectures with respect to the uses of the several constituents of the blood in the animal œconomy, and stating some of the changes in colour which various chemical agents produce in it, we are next introduced to the subject of the *coagulation of the blood.*

" The separation of crassamentum from serum is ascribed to the contraction of the fibrine; for if this constituent be removed immediately on the effusion of the blood, no coagulation takes place. When we consider the small proportion of fibrine in blood, viz. 2·4 parts in 1000, we are surprised at the phenomenon. Is not coagulation rather an effect of the attraction of hæmatosine and albumen? In the living circulation, the action of an affinity may be prevented: deprived of vital influence, the blood, like every other fluid of the animal system, becomes subject to chemical laws." 53.

The above is not the only instance in the book wherein the author makes sad work, when he attempts to philosophize. For further information on this subject, see the April number of this Journal of the present year, p. 501. From a number of experiments undertaken with a view to determine the arrangement of the fibrine, and the proportion which exists in different parts of the crassamentum, Mr. Thackrah concludes that there is *most fibrine in the lowest part of the crassamentum, and least in the middle.* These experiments also would make it appear that the hæmatosine and albumen arrange themselves as variously as the fibrine.

That heat is evolved during coagulation has been affirmed and denied. Hunter and Dr. John Davy have decided in the negative, whilst Fourcroy, Dr. Gordon, and others have maintained the affirmative. Several experiments were instituted on the subject by the author, in which no rise of the thermometer was observed at the moment of coagulation.

Some observations made by Sir E. Home and Mr. Brande favour the opinion that carbonic acid gas is evolved during the coagulation of the blood. Dr. Davy denies it. Sir C. Scudamore contends that experiments instituted by him establish the fact. "If we acknowledge the existence of a free alkali in the blood," says our author, "we cannot conceive the co-existence of an acid gas." One of the reasons given by Dr. Davy in support of his opinion is, that he has added one-fourth of a cubic inch of carbonic acid gas to an ounce of blood, and to a similar quantity of serum, "the whole of which has been absorbed, and yet the blood and serum still exhibited free alkali." The editor, Dr. Wright, very pertinently asks, "Do not these experiments prove that the free alkali exists in some state in which it is not acted on by the presence of carbonic acid? If so, they invalidate the objection urged by Mr. Thackrah in the next sentence." Dr. Stephens maintains the existence of carbonic acid in the blood;—he says that this acid is attracted and carried off by the oxygen of the atmosphere before the blood can be subjected to such experiments as those stated by our author. An experiment performed by Dr. A. T. Thomson, and detailed in the work by the editor, is to the same purport: as are also some experiments by Dr. Clanny, given in the Edinb. Journ. XXXII. 40. See also Brande, in Phil. Trans. 1818, 181, and Vogel, in *Annals of Philosophy*, VII. 57. Dr. Prout believes that one copious source of this acid in the blood is the conversion of its albumen into the gelatinous secretions of skin, &c.; gelatin containing three or four per cent. less carbon than albumen.*

Coagulation is influenced also by the quantity of blood in relation to the surface over which it is spread. The rapidity of coagulation being inversely as the quantity of blood, and directly as the extent of surface. The material and shape of the vessel also seem to influence the quickness of coagulation. Dr. Babington found the proportions of serum and crassamentum to vary materially in the same blood drawn into differently-shaped vessels.† The author instituted some experiments in reference to the shape and material of the vessel, and suspects that the curious diversities with respect to the time of concretion, and the resulting proportions of serum and crassamentum to depend on the electric conditions of the respective metals in which the blood was contained. It is to be regretted that he did not make himself more intelligible with respect to the nature and grounds of this his suspicion.

Coagulation appears to be influenced by temperature also. According to Hewson and Hey,‡ the temperature of 98° is most favourable to coagulation.

According to our author, a temperature of 120°—130°, accelerates very considerably the concretion of the blood, and one of 100°—110° generally does so, but not in so marked a manner.

"When blood, on the point of concretion, is placed in a freezing mixture, the natural action is suspended, and a few drops only of serum exude.

---

* Bridgewater Treatise, No. VIII. 524.

† See Medico-Chirurg. Trans. xvi. pt. 2, 296-7.

‡ Exp. Inquiry, p. 6. Obs. on the Blood, p. 38.

How shall we account for the prevention of coagulation by cold? does cold prevent the play of chemical affinities?" (68.) Before the author proposed this question, he should have shewn, or endeavoured to show that coagulation arises from the play of chemical affinities. The author next introduces experiments to shew that moderate agitation seems to promote concretion.

In the next chapter we are introduced to a very interesting subject, namely, the *cause of the blood's coagulation.* Various hypotheses have been employed to account for this result. Some have thought that the fluidity of the blood depended on the heat of the body, and that its coagulation is produced by its removal into a colder temperature. Experiments have proved the weakness of this hypothesis. From cases related by Morgagni, the observations of Fontana, and numerous experiments instituted by the author himself, he shews satisfactorily, that "the vital or nervous influence is the source of the blood's fluidity, and its loss the cause of coagulation." (91.) Whether this vitality resides in the blood itself, he leaves for others to consider. It would appear to us, that he is rather disposed to place this vitality in the vessels.

In the next chapter, the author states the results of his experiments on blood from different vessels of the same order. And, first, with respect to jugular and caval blood, taken from dogs, as nearly as possible, at the same time and under similar circumstances; the conclusion he arrived at, with respect to their coagulation, was, that "CONCRETION almost always takes place sooner in blood from the vena cava than in that from the jugular." 95. With respect to the SOLID CONTENTS, he always found them in larger proportion in jugular than in caval blood. From this difference in the contents of the jugular and caval veins, he was led to extend his enquiries to other vessels, and particularly with respect to the *vena portæ.* The contents of this vessel were found to be darker in *colour* than blood from other veins. Portal blood, also, was observed not to have the homogeneous character of other blood, its appearance giving the idea of imperfect elaboration. With respect to *specific gravity,* there was not found to be any marked difference in portal blood. With respect to the *comparative periods of concretion,* portal blood was observed to concrete much sooner than blood from other veins.

With respect to *the ultimate proportions of serum to crassamentum* in portal, as contrasted with jugular blood, experiments led him to the inference, that portal blood contains from about $\frac{1}{10}$ to $\frac{1\frac{1}{2}}{10}$ more serum than blood from other veins; and, also, that the "separative change is much slower and less perfect in portal than in jugular blood."

The *character of the serum* of portal blood differs from that of jugular, the former being always observed to be red, and the latter straw-coloured. In order to account for this an experiment was instituted, the inference from which was, that the colour of portal serum depends rather on the state of the crassamentum in portal blood, than on any power possessed by portal serum of holding the red particles in solution. With respect to *the coagulation of portal serum* by heat, it was found to be less perfect than that of jugular serum, which our author supposes to depend on a difference in the state of the albumen, and not on the detention of the red particles, as may appear at first view.

The *disposition to putridity, in portal serum,* he found to be less than in jugular serum.

To ascertain *the proportion of solid matter in the serum* of portal, as contrasted with that in the serum of jugular blood, on experiment, the average was found to be in favour of portal serum containing more solid matter than jugular. This, however, subsequent experience inclined him to think attributable rather to the detention of the red particles, than to a larger proportion of albumen. With respect to the *crassamentum* in portal, contrasted with jugular blood, it was looser in texture than jugular, and contained serum up to the period of putrefaction. Our author's experiments on portal and jugular blood led him to the following inferences.

1. "That the blood from the vena portæ has the appearance of defective elaboration, and that its colour is darker, and more inclined to brown than to the modena."

2. "That portal blood concretes more quickly than blood from other veins."

3. "That it contains much more serum; and, 4, that the serum of portal blood, from the detention of colouring matter, is redder than serum carefully separated from the blood of other vessels. 5. That portal serum, by heat, concretes more quickly, but less completely, than jugular. 6. That the crassamentum of portal blood does not expel its serum as fully as blood from other vessels. 7. That portal crassamentum contains a smaller quantity of fibrine; and, 8, that portal blood in general contains much less albumen and hæmatosine than jugular." 111.

### CHAP. VI.

In this chapter, which has been added to the present edition by the editor, are noticed *the differences in the physical and chemical constitution of arterial and venous blood.*

In the physical characters of arterial and venous blood, the following distinctions have been observed.

"1. In COLOUR. It is well known that arterial blood is generally of a florid scarlet hue, and venous of a Modena purple. This difference is most striking in the vessels near the heart; but it is also observable in the minutest capillaries. It does not, however, uniformly exist. In the *puer ceruleus,* for instance, a portion of venous blood passes to the left side of the heart, without having been transmitted to the lungs, and the blood in the arteries has a tinge of purple; and in those diseases in which respiration is imperfect, there is a similar result. Mr. Vines has observed, that if the spinal marrow of a horse or ass be divided as close to the brain as possible, the moment respiration ceases, the arterial blood becomes as dark-coloured as venous, and of the same temperature. Again, venous blood often approaches in colour to arterial, especially under great excitement." "The cause of this difference in colour is a question which has not yet been satisfactorily solved. The old opinion, that it arises from oxygenation of the ferruginous envelopes of the globules, has given way before the test of chemical analysis; and various other ingenious hypotheses have shared the same fate. Of those which are now advocated, the most generally received is that which ascribes the alteration to a decarbonization of the venous blood, and to a slight increase in the temperature of the arterial. Dr. Stephens has endeavoured to prove, that the arterial colour is produced by a change in the saline ingredients of the blood, and has quoted experiments, in which a florid scarlet hue was given to crassamentum by dipping it in an artificial saline serum, and not by oxygen gas." 116.

Dr. Turner was induced, by experiment, to adopt Dr. Stephens' opinion,

M. Barrueil, in the Annales d'Hygiène, April, 1829, p. 269, states, that blood kept for several weeks still preserves the property of being changed to a vermilion colour by oxygen gas, even when some of its elements, and particularly the fibrine and albumen, have undergone decomposition. It appears to him, that the colouring matter of the blood, on which the oxygen acts in preference, is endowed with great vital force, which is not extinguished till a considerable time after the complete death of all the other immediate principles of the same liquid. In support of this latter view, Berzelius, in his "View of Animal Chemistry," states that blood which still contains the colouring matter absorbs oxygen gas very quickly, when out of the body, and shaken in atmospheric air; on the other hand, serum, when destitute of colouring matter, does not change the atmospheric air before it begins to putrify.

"The *quantity* of blood is much greater in the veins than in the arteries, In addition to their functions as circulatory vessels, they perform the office of reservoirs, through which the current passes more or less languidly, according to the demands made on them by the heart and arterial system. Red blood is more abundant during youth than in manhood or old age, and is supplied most abundantly to those organs which are in progress of growth; in all cases, however, the dark blood preponderates." 116.

With respect to *temperature*, there is considerable discrepancy in the statements of authors on this point; in consequence of its close connexion with the subject of animal heat, great attention has been directed to it..

"Crawford asserts that, in the pulmonary vessels, arterial blood possesses a larger amount of absolute heat than venous. The average deduced from his experiments is, that the capacity for caloric of the fluid (arterial), in the pulmonary veins, is to that (venous) of the pulmonary arteries, as 97·08 : 112, or nearly as 10 : 11½. Majendie computes it at 852:839." "Majendie states 101·75° to be the mean grade of venous blood, and 104° that of arterial." 117.

With respect to the *specific gravity*, there is no uniformly marked difference to be observed; *coagulation* is said by some to take place more rapidly in arterial than in venous blood, and that the former has a smaller proportion of serum.

### Chap. VII.

In this chapter are noticed the *Effects of States of the Animal System on the Blood*. And with respect to *temperature* of body; this, being subject to such slight variation, can produce very little change in the character of the blood. The blood is said, however, to be darker in cold regions than in temperate ones. *Age* has considerable effect on the blood, its quantity being greater in youth than in advanced life; at an early period, also, it is observed to be bright in colour, coagulates quickly, throws off little serum, and leaves the crassamentum soft and watery. With respect *to sex*, our author observes that there is generally found more water and less fibrine in the blood of females than in that of males; he conceives this to arise from their different habits of living. *Muscular exercise*, he observes, has a marked influence on the character of the blood—it appears to heighten its scarlet hue.

Digestion and diet have great effect on the blood. It is evident, if the process of digestion be incomplete, the quantity and quality of the chyle, and consequently that of the blood, must be materially affected. From some experiments made by the author on dogs which had fasted, and on others which had been fed recently, it appears "that blood from the fasted animal does not so quickly concrete; that its serum contains a proportion of albumen about equal to that from one recently fed; that its crassamentum yields rather more hæmatosine, albumen, and fibrine; and, as a consequence of all these, rather less water." (128.) The principal difference he observed to consist in the period of concretion, to which there is a greater disposition in blood from a recently-fed animal.

He next considers, *whether the milky or cream-like appearance, which serum sometimes assumes, depends on digestion.*

This phenomenon has given rise to much conjecture. Hewson conceived this substance to be produced by the absorption of fat. Dr. Marcet seems to think the substance to be derived from the chyle of animal food, and that it is closely allied to cream; and Berzelius states that a portion he examined consisted of this fluid and albumen; whilst Raspail, in his Organic Chemistry, considers the phenomenon to be produced by the presence of an acid in the blood, which saturates the alkaline menstruum of the albumen, and hence it is precipitated from the serum. He remarks, this effect may be produced by excess in the use of spirituous liquors, or by inflammatory action.

The *quality* of the food influences the blood, vegetable diet diluting, and animal thickening it. This observation, however, as our author remarks, applies only to the hæmatosine and water, but not, as far as he thinks, to the proportion of albumen. He states, also, that cellular dropsy, without diseased liver or ascites, has been frequently observed to arise from the continued use of poor, low, and vegetable diet, whilst, on the other hand, excess of animal food, without proportionate exercise, seems to reduce too much the aqueous part of the blood, thereby giving rise to an inordinate secretion of uric acid from the kidneys, and gravel; frequently, also, to concretions on the joints. *Fatness* or *leanness* also seem to affect the quantity and quality of the blood, fat animals having in general less of it, in proportion to their weight, than lean ones.

*Impressions on the nervous system* have a remarkable effect on the blood.

"Syncope immediately disposes the blood to concrete. In venæsection, when this process has commenced in five minutes, faintness has reduced the period to two; and when ninety seconds were before required, deliquium has instantly caused the blood to cake in forty." 132.

With respect to the effects produced on the blood by the state of the system, in reference to *strength* or *debility*, various and opposite opinions have been entertained with respect to the period of its concretion. From numerous and careful experiments instituted by our author on this point, he infers that, "in the dog, sheep, horse, and hog, the blood concretes slowly, in regular proportion to the tonic state, or that condition of the system in which the vital powers are strongest." With respect to the exudation of serum, as influenced by debility, he uniformly found that "blood, taken from an animal in articulo mortis, never fully separates its serum, and rarely throws off even a small quantity."

After stating that *hæmorrhage increases the tenuity of the blood*, an opinion, by the way, held universally by the profession, our author asks, "how shall we explain this fact? Shall we consider that a sensation of inanition is produced by copious bleeding in the blood-vessels, and the whole corporeal system, and that, in consequence, the provident principle of nature excites the absorbents to increased action?" The author might have recollected that it is an opinion universally entertained by medical men, that loss of blood excites the action of the absorbents, and in fact that when the physician wishes for example to ensure and expedite the action of mercury on the system, he possesses an infallible means of doing so by having recourse to venæsection. It is an admitted principle, that lowering the action of the heart and arteries in any way excites the action of the absorbents, and it is with this view that digitalis is given in dropsy.

From some experiments instituted by the author he concludes that "utero-gestation increases the proportion of albumen and hæmatosine." 148.

We shall now pass on to Chap. IX. which treats of the BLOOD IN DISEASE.

SECT. 1. *Observations on the Blood in its Vessels.*

"Blood is sometimes found strongly coagulated in its vessels. Haller remarked a concreted tremulous jelly in the veins, even of a living person. Limited coagulation is often seen in mortification. It is also found, though to a minor extent, above the ligature or division of an artery. But the part in which the appearance is most remarkable is the heart. In this organ considerable masses of white or rose-coloured coagula (false polypi) are not unfrequently found after death, attached near the large valves." "The opinion common in the profession that false polypi are formed after life is extinct, and have consequently no etiological importance, is opposed by the examination of their structure." "Death, therefore, I conceive, in a great number, perhaps in a majority of cases, has polypi for its *immediate* cause." 165.

Our author next discusses the fluidity of the blood in its vessels, and on this subject he gives a large quotation from an essay on cholera. He here combats the opinion of the non-coagulation of the blood in this disease, and states his suspicion that most of the cases recorded as such were founded only on retardation of the process, in as much as when this fluid is removed out of its natural vessels, signs of concretion become soon apparent. He offers an ingenious conjecture by way of accounting for this fluidity after death in such cases. In all cases of death, where the nervous system has been the part primarily attacked, as in fatal impression from lightening, certain poisons and accidents, and in fatal cholera, he supposes that life remains for a considerable time in the blood-vessels—certain it is that irritability remains for a long time in the muscles. The author conceives that too much importance has been attached to the fluidity of the blood in its vessels, it being generally considered an evidence of violent death.

"Morbid productions and natural substances from other parts of the body are sometimes found in the blood-vessels. Purulent matter has been noticed in the veins, and sometimes also in other parts of the circulatory system. Pus has been known to circulate in the human frame." "Portal and Dupuytren have seen pus in the lymphatics surrounding an abscess." "Independent of the direct evidence of pus in the blood-vessels, we have indirect though not less decisive proof. The practice of surgery not unfrequently shews the removal of an abscess without external evacuation, and the deposit of matter far from its original seat."

"Besides the morbid states of blood, which are observable in its vessels, there are others of a peculiar nature known only by their effects. The blood appears to become actually poisonous." "Veterinary surgeons have propagated disease from horse to horse by the transfusion of blood." "In this way the fatal catarrh, called glanders, has been transmitted by Professor Coleman, and the 'malignant pustule' by Dupuy and Leuret. According to Doctor Hertwich, of Berlin, the blood of a rabid animal will, by inoculation, communicate the disease."

The effects produced on the system by various substances injected into the blood are then noticed. Air, when admitted into the circulation in considerable quantity, has caused death. Majendie has related some cases of this. Bichat injected into the blood ink, oil, wine, water coloured with indigo, and other substances. These, when received by the crural artery, produced torpor, sometimes paralysis but not death. Alcohol, when injected into the blood in large quantity, has produced death.

"More remarkable and important are the phenomena produced by the injection of pus or fetid matter into the circulation. On this point M.M. Gaspard and Majendie have made some very interesting statements. The former first injected into the jugular veins of dogs pus diluted with water. They became immediately agitated, made efforts at deglutition, then sunk faint, moaned and vomited. The bladder and intestines were emptied. Recumbent on the side, with respiration imperceptible, and pulse very feeble; they at length voided fæces liquid and extremely fetid. This afforded great relief, and either procured a speedy restoration of health, or was succeeded by dysenteric symptoms, exhaustion and death. When he encreased the quantity of pus injected, the nervous symptoms were sooner and more strongly marked, wanderings of the eyes, excessive sensibility, involuntary startings, hiccough, convulsions, and delirium. In one case a sort of emprosthotonos with stiffness of the limbs ensued at the end of fifteen minutes, on the injection of three drams of pus. Post-mortem examination exhibited, in less urgent cases, nothing remarkable, with the exception in one of partial hepatization of a portion of lung." 175.

Our author is inclined to think that the principal impression, in all these experiments, was on the nervous system, and through it on the muscular system of organic life. He next notices the effect on the blood of certain articles taken by the stomach. Prussic acid is found to render the blood florid, and to hasten its concretion. Mercury is said by some "to possess the power of breaking down the crasis of the blood." Our author never observed such an effect produced by this mineral. Alimentary substances of bad quality, or deficient in quantity, produce a vitiated state of the blood; thus the use of herbs and uncooked roots among the poor has been found to produce dropsy. Scurvy is produced in crews by the exclusive use of salted provisions.

Our author having considered the principal changes observed in blood in its vessels, now turns his attention to those changes which analysis exhibits in it when obtained by venesection, in disease. He here makes some pertinent remarks regarding the important part performed by the fluids in disease, and the necessity of cultivating their pathology. "The *colour* of the blood in disease is of considerable importance. Its deep rich colour is reduced by hemorrhage, for hæmatosine appears to be of less easy reproduction than the other constituents of blood." "Baglivi observed that the blood of venæsection had a bright scarlet hue in hectic patients." "Cholera, in its purple form, shews the darkened and depraved state of the blood."

"The *temperature* of the blood has been changed in some cases of disease. In fevers and internal inflammations, though the thermometer is not generally raised above 97°, many instances have occurred in which it has been elevated to 104°, 107°, and even 110.°" The *specific gravity* of the blood is not much changed in disease. With respect to *coagulation*, the rapidity with which blood concretes is found to be proportioned to the debility of the individual—and this circumstance, as our author well remarks, is of considerable importance in a curative point of view, the first natural check to hæmorrhage being the formation of a clot on the mouth of the vessel.

From some experiments made to ascertain the effects of a tonic and atonic state of the system on the concretion of the blood, the author feels himself warranted in propounding the following opinion: "that the speedy occurrence of concretion on the effusion of blood, affords a reason sufficiently cogent for the discontinuance of depletory measures." Several pathologists have recorded cases of disease in which the blood when obtained by venæsection did not present any sign of coagulation whatever. Our author doubts the accuracy of these accounts.

"The *firmness of the coagulum* of blood has been considered a distinctive mark of a tonic state of the system; its great tenacity, a characteristic of inflammation; and its looseness, a sure proof of debility." 192.

"As the density of the coagulum has had a considerable effect in the treatment of disease, I shall advert to two or three points of fallacy on this subject. It is frequently found that serum is slowly exuded; and hence unless a due time elapse before examination, the coagulum is soft from the serum it contains. Here, upon the general principle, the practitioner would desist from further evacuations, concluding the system to be greatly reduced. Sometimes, also, from the adhesion of the coagulum to the side of the vessel, from the kind of vessel, or other causes, the separation of serum is prevented for many hours, yet, on the removal of such attachment, or on the division of the coagulum, the serum is effused, and the crassamentum becomes firm." "If, however, on the division of the coagulum, at the expiration of from eight to twenty-four hours, there ensue no considerable effusion of serum, and the crassamentum remain extraordinarily firm, I believe that further depletion is fully warranted." 194.

The *proportions of serum and crassamentum* are also found to be considerably affected by disease. Our author's experiments incline him to state, that "acute disease reduces the proportion of serum;—in other words, increases the mass of crassamentum."

Considerable importance has been attached to the BUFFY COAT. It has been usually considered as an infallible criterion of the existence of inflammation.

"In some cases the surface is concave, and this cupped appearance is greatest, I think, when the quantity of blood is small. In blood of a buffy constitution, the formation of the tunic is considerably affected by the mode in which the fluid is abstracted. A small trickling stream will prevent the appearance of the sizy tunic. The kinds of vessel in which the blood is received will also have an effect in altering its character." 201.

For some very interesting experiments on the subject made under the direction of Professor Recamier at the Hôtel Dieu, see this Journal for 1824. The buff-coat is generally observed in blood drawn during pregnancy. It is seldom observed in mucous inflammations. Some medical men think that the colour and figure of the buff are characteristics of the seat of the disease.

"We have found great diversity in the solid contents of the blood. Its thickness is sometimes diminished, but much more frequently increased." "The proportion of solid contents in the blood is remarkably increased in almost all those diseases for which venæsection is prescribed." 208.

"The quantity of fibrine has been considered to bear a proportion to the acute character of the disease." "The quantity of fibrine bears probably a relation rather to the extent and nature of disease than to the state of the constitution. Where there is increased action without reduction of power, the fibrine, I believe, is not increased; but where these circumstances are conjoined, it is. Hence in second and third bleedings of the same patient, the blood frequently contains more fibrine than the first." 210.

We have been thus copious in our analysis of this work. The vast importance of the subject of which it treats, and our conviction of the great advantage to be derived to medicine from due attention being paid to this branch of animal chemistry, will serve as our apology, if apology were necessary. To the lamented author the profession is much indebted for having contributed this his mite to the illustration of the pathology of the fluids. The manner in which the work is executed, evinces no ordinary degree of patient industry. Whatever inaccuracies or deficiencies may be found in it, would of course have been corrected, had the writer been spared to revise and superintend this edition. We must not however let this opportunity pass without expressing our approbation of the manner in which the editor Dr. Wright has performed his part. The judicious annotations given by him enhance considerably the value of the book. We therefore feel no hesitation in recommending it to the perusal of our medical brethren.

